# Advancements in robotic assisted microsurgery: Exploring the MUSA robot in reconstructive microsurgical and supermicrosurgical procedures

**DOI:** 10.1016/j.jpra.2025.11.033

**Published:** 2025-12-04

**Authors:** Hendrikje Bettens, Raphael Timmerman, Shan Shan Qiu, René van der Hulst, Tom van Mulken

**Affiliations:** Department of Plastic, Reconstructive, and Hand Surgery, Maastricht University Medical Center, Maastricht 6229HX, The Netherlands

**Keywords:** Robotic assistance, Microsurgery, MUSA2, MicroSure

## Abstract

Robotic assistance in microsurgery offers increased precision, tremor elimination, motion scaling, and improved ergonomics. Although robots are widely used in other surgical fields, their integration into reconstructive microsurgery has been limited. The MUSA robot is the first robot specifically designed for microsurgery to address this unmet need. This report presents a comprehensive overview of the clinical indications and experiences with the MUSA2 robot at our center, including lymphatico-venous anastomosis, free flap breast reconstruction, free flaps reconstruction of the lower extremity, digital nerve repair, and finger replantation.

Robotic assistance has great potential for its use in microsurgery. Challenges such as higher cost and setup times could be addressed by the evolution of the systems. Due to its compatibility with standard instrumentation and short learning curve the MUSA robot has promising initial results. This stimulates further research into robotic assistance as a tool in complex microsurgical procedures.

## Introduction

Since the first robotic-assisted anastomosis in reconstructive microsurgery was performed in 2006, there has been a growing interest in the use of robotic assistance in microsurgery.^1^ Robotic assistance offers several advantages in microsurgery, with a common feature to overcome the limitations in precision of the human hand through motion scaling and tremor filtration. It takes an extensive amount of training to master microsurgery and not all surgeons are able to master these precise and delicate movements.^2^ A recent study by Friedberg et al. showed that the learning curve for robotic-assisted micro anastomosis is steeper than for manual anastomosis, regardless of previous (micro)surgical training,^3^ suggesting that the use of robotic assistance may lead to a more efficient development of microsurgical skills. Furthermore, robotic systems contribute to improved ergonomics of the microsurgeon.^4^ Reconstructive procedures are often lengthy procedures, requiring surgeons and assistants to operate in uncomfortable working positions. Robotic assistance can enhance ergonomics and reduce such physical strain and fatigue, improving the overall quality of the procedure.^5^

Since the introduction of the da Vinci robotic system, robotic assistance has been extensively integrated and applied in various surgical disciplines, including urology, gynecology, and gastrointestinal surgery.^6^^,^^7^ In contrast to the theoretical advantages of robotic assistance in microsurgical operations, the adoption has been slow in this field. This is partly because the available robots were not developed specifically for microsurgery and therefore lack the specific requirements for this delicate work. In 2014 a collaboration between Eindhoven University of Technology (TU/e) and Maastricht University Medical Centre (MUMC+) called MicroSure, created the first robot specifically designed for (super)microsurgery. This robot was called MUSA (Micro(μ) Surgical Assistant).

Preclinical studies using the MUSA robot in silicone vessels and rats have demonstrated the technical feasibility for microsurgical anastomosis.^3^^,^^8^^,^^9^ The first clinical study demonstrated the clinical feasibility and safety of the MUSA2 robot for robot-assisted supermicrosurgery (anastomosing vessel diameters between 0.3 and 0.8 mm diameter). This prospective randomized study compared manual versus robot-assisted lymphaticovenular anastomoses (LVA) in the treatment of breast cancer-related lymphedema and concluded comparable clinical results in both groups after 1 year, indicating clinical safety and highlighting the potential of robotic systems for the field of reconstructive microsurgery.^10^^,^^11^ The potential of robot-assisted microsurgery and supermicrosurgery with the MUSA robot is further explored in clinical pilot trials in our institution.

Although robotic assistance is increasingly used in surgical procedures, the adoption of robotic-assisted microsurgery is limited. This report aims to discuss the various indications for which robotic assistance with the MUSA2 robot is used in microsurgery in our center with description of various cases. This ranges from the common microsurgical procedures, such as free flap arterial anastomoses, to more subspecialized and supermicrosurgical procedures, including digital replantation and lymphaticovenular anastomoses. Future perspectives and potential applications of robotic assistance in microsurgery are provided.

## Indications for robotic-assisted microsurgery

All patients provided written informed consent prior to treatment. Additionally, each patient signed a separate consent form granting permission for the use of clinical images for scientific publication purposes.

### Lymphedema reconstruction

A 55-year-old patient presented to the lymphedema clinic with lymphedema in her left arm following treatment for breast cancer. Her oncological treatment, completed 6 years prior to presentation, consisted of a mastectomy and axillary lymph node dissection, followed by chemotherapy. At the time of evaluation, the lymphedema was classified as stage IIa according to the International Society of Lymphology.^12^ Indocyanine green lymphography revealed dermal backflow at stage III, with suitable lymphatic vessels for lymphaticovenous anastomoses.^13^

The patient underwent three anastomoses at the wrist level, performed using the MUSA2 robot. The procedure was completed without complications. At 1-year follow-up, the patient reported a reduction in swelling and symptoms, which was consistent with an improvement in her LYMPH-ICF score (decreasing from 52 preoperatively to 10 postoperatively).

A comparable case involved a 61-year-old woman with lymphedema following breast cancer treatment, which included mastectomy and axillary lymph node dissection, along with adjuvant radiotherapy. She presented at the lymphedema outpatient clinic due to frequent recurrent episodes of erysipelas, occurring several times per year. Indocyanine green lymphography revealed stage III lymphedema, with suitable lymphatic vessels for lymphaticovenous anastomosis.^13^ Using the MUSA2 robot, two anastomoses were performed at the wrist level. At 1-year follow-up, the patient reported high satisfaction with the outcome, which was consistent with the LYMPH-ICF score: 41 preoperatively, and 16 and 19 at 1 and 2 years postoperatively, respectively. Additionally, there was a reduction in the frequency of erysipelas episodes.

### Lower extremity free flap reconstruction

A 58-year-old male patient underwent a flow-through anterior lateral thigh (ALT) flap following wide excision of a calcaneal melanoma (pT3bN0M0 acrolentiginous melanoma). The posterior tibial artery and vein were used as recipient vessels, with a venous end-to-end and an arterial flow-through anastomosis. Robotic assistance of the MUSA2 robot was used for the arterial anastomosis. The total ischemia time was 54 min of which 28 min were for the making of the arterial anastomosis. The patient healed well, with a minor wound dehiscence, which was managed conservatively.

Another case involved a 35-year-old man with a soft tissue defect following re-osteosynthesis for a pilon fracture. A superficial circumflex iliac artery (SCIP) flap was used for the reconstruction. One vein was anastomosed using a microvascular coupling device (GEM Coupler, Synovis Micro Companies Alliance, Inc., USA), while a second vein was anastomosed manually with 9-0 Ethilon® sutures (Ethicon, Johnson & Johnson, USA). The arterial anastomosis with robotic assistance of the MUSA2 robot, was completed in 24 min, with a total ischemia time of 70 min. The procedure and post-operative follow-up was without complications.

### Microsurgical autologous breast reconstruction

A 60-year-old woman underwent a secondary deep inferior epigastric perforator (DIEP) flap reconstruction with robotic assistance. She had a history of right-sided breast cancer treated with mastectomy, chemotherapy and hormone therapy. Magnetic resonance angiography identified an optimal perforator on the left side, which was selected for the DIEP-flap breast reconstruction. The arterial anastomosis was performed end-to-end with the MUSA2 robot and 9-0 Ethilon® sutures (Ethicon, Johnson & Johnson, USA). The ischemia time was 65 min (53 min dedicated to the arterial anastomosis). The procedure and use of the MUSA2 was uncomplicated. Besides some hypertrophy of her scars, follow-up at the outpatient clinic was without problems up at 1-year follow-up.

### Digital nerve repair

A 34-year-old man presented to the emergency department with a laceration to the radial side of his second digit, suspected of radial digital nerve injury. No other injuries were identified. Surgical exploration confirmed a complete transection of the radial palmar digital nerve at the level of the proximal interphalangeal (PIP) joint . Microsurgical repair was performed with an end-to-end anastomosis, using robotic assistance via the MUSA2 robot and 9-0 Ethilon® sutures (Ethicon, Johnson & Johnson, USA) under local anesthesia in an outpatient treatment setting. Postoperative follow up at 12 months showed a full range of motion of the hand without complaints of residual sensory loss. The static two-point discrimination testing at 12 months follow up showed mean distance to the affected finger of 4 mm and the non-affected finger of 3 mm, both these values correspond to a good score.^14^

A similar case involved a 31-year-old woman who sustained a partial digital nerve injury of her index finger, while cutting an avocado. Surgical exploration revealed a partial transection of the ulnar palmar digital nerve at palmar level. The nerve repair was performed with robotic assistance under local anesthesia in an outpatient clinic setting, and the patient recovered with full range of motion of the finger and minimal residual sensory loss. At the 12-month follow-up, static two-point discrimination testing showed an average minimum distance of 5 mm for the affected finger and 2 mm for the unaffected finger, with both values indicating a good outcome.^14^

### Digital replantation

A 47-year-old man sustained a complete thumb amputation at the metacarpophalangeal joint due to a circular saw injury. Replantation was performed under general anesthesia, with metacarpophalangeal joint arthrodesis for bony fixation and conventional flexor tendon and extensor tendon repair. Robotic-assisted arterial anastomosis was performed with 10-0 Ethilon® sutures (Ethicon, Johnson & Johnson, USA) sutures and two dorsal veins were anastomosed with a 1.0 mm microvascular coupling device (GEM Coupler, Synovis Micro Companies Alliance, Inc., USA). Due to insufficient arterial flow the arterial anastomosis was revised and the surgeon performed this manually including the nerve anastomoses to reduce time of surgery. The patient was discharged after 6 days, with a vital and functional thumb at 12-month follow-up .

## Discussion

This article provides a comprehensive overview of the various clinical indications for which robotic assistance in microsurgery is utilized with the MUSA2 robot in our center.

Lymphatico-venous anastomosis is a supermicrosurgical procedure requiring high precision. Van Mulken et al. demonstrated the feasibility and safety of robotic assisted lymphatico-venous anastomosis using the MUSA robot and reported no statistically and clinically significant differences between robotic and manual lymphatico-venous anastomoses in a prospective randomized pilot study.^10^^,^^11^ Other studies focusing on robotic lymphatico-venous anastomoses confirmed its feasibility but did not report patient outcomes.^15^, ^16^, ^17^, ^18^

In the field of DIEP-flap breast reconstruction, recent advancements have been made regarding the integration of robotic assistance into various steps of the procedure. Robotic-assisted endoscopic flap harvesting has been evaluated using the DaVinci robot (Intuitive Surgical Operations, Inc., Sunnyvale, Calif.) for reduction of donor site morbidity.^19^ In addition to robotic-assisted endoscopic harvesting of the DIEP flap, the use of robotic-assistance for performing the anastomoses has also been explored. Several studies have demonstrated the feasibility of this approach with the Symani Surgical System.^20^^,^^21^ Our experience with the use of the MUSA2 robot, supports the feasibility of robotic assistance for the microsurgical anastomosis in DIEP flap breast reconstruction and underscores the promising role of robotic assistance in various steps of DIEP flap reconstruction. In our experience, intraoperative movement of the anastomotic site at the internal mammary vessels, caused by patient respiration, can complicate the procedure when using current robotic systems. In conventional microsurgery, the hand of the microsurgeon rests directly on the chest wall which compensates for this respiratory motion. Robotic systems for microsurgery lack this stabilizing feedback. We have observed a variance in respiratory motion among patients and have addressed this issue by reducing the tidal volumes during surgery. In our opinion, future developments in robotic systems and techniques will have to adapt to this moving operating field.

In our series of free-flap lower extremity reconstruction, several types of free flaps were performed (SCIP-flap, ALT flaps). In this ongoing prospective study, no flap-related complications have been encountered. This favorable outcome is consistent with findings in the existing literature. A study by Struebing et al. examined 39 free flaps for lower extremity reconstruction with robotic assistance of the Symani Surgical System, involving 13 arterial and 36 venous anastomoses. The reported anastomoses were successful except for one flap loss (2.56 %), of which the cause was not further specified. One other revision was performed manually for arterial thrombosis.^22^ Furthermore, a study by Mori et al. supports the feasibility of robotic assistance in lower extremity reconstruction. In this study, 40 microvascular anastomoses were performed with robotic assistance of the Symani Surgical System, including 16 arterial and 24 venous anastomoses, all of which remained patent. One flap developed partial necrosis due to venous insufficiency.^23^ When comparing these outcomes to conventional manual free flap reconstruction of the lower extremity, a systematic review in 5061 patients reported 7.78 % flap failure which is comparable to the initial robotic outcomes.^24^

The outcome of microsurgical repair of transected nerves is based on many patient and trauma related factors besides the technical quality of the repair. However, robotic assistance has the potential to enhance the quality of nerve coaptation.^25^ In 2012, Lequint et al. demonstrated the feasibility of minimally invasive robot-assisted surgery of the brachial plexus by performing a biopsy of an intraneural perineurioma using the Da Vinci S® system, resulting in a successful diagnosis and the absence of postoperative sensory or motor deficits.^26^ In 2023, Schäfer et al. reported a case of robotic-assisted nerve coaptation using the Symani Surgical System, demonstrating the potential application of robotic technology in peripheral nerve repair.^27^ Another study on peripheral nerve reconstruction, conducted by Aman et al., explored various nerve surgeries performed with robotic assistance, including nerve transfers, targeted muscle reinnervation, neurotized free flaps, and autologous nerve grafts.^28^ In the study by Struebing et al., 31 nerve coaptations were performed, with one case of hematoma requiring evacuation.^29^ While these studies demonstrate the feasibility of nerve coaptation using robotic assistance, they do not provide data on functional outcomes. Our prospective studies using the MUSA2 robot for digital nerve repair evaluates clinical outcomes and these results are to be published in the near future. Combining developments in supermicrosurgery, vascularized nerve grafts and fascicular nerve repair, robotic-assistance might open new doors to better patient outcomes in peripheral nerve surgery.

There is considerable discussion regarding the indications and contraindications for finger replantation. In general, thumb amputations, multi-finger amputations, and transmetacarpal amputations are considered appropriate indications.^30^ In contrast, replantation of a fingertip amputation is more controversial since the blood vessels are very small and difficult to access.^31^ In this context, the use of robotic assistance may offer potential advantages. However, to date, there are no studies evaluating robotic-assisted replantation of fingertip amputations. A study by Dastagir et al. evaluated the use of robotic assistance for vascular anastomoses in finger injuries, including replantation, and compared it to manual anastomoses. Their results confirmed the feasibility of robotic-assisted anastomoses without specifying the level of amputation.^32^

Robotic assistance in microsurgery remains an emerging field. A growing amount of studies have explored the feasibility of several robotic systems. Critical evaluation of its clinical utility, especially in terms of outcomes and efficiency, is ongoing. The cases described in this series confirms the feasibility of robotic-assisted microsurgery using the MUSA2 robot for several indications. While there are many positive findings, there are still limitations associated with robotic assistance. The most reported disadvantage is the lack of haptic feedback.^3^^,^^4^^,^^33^ In a study by Besmens et al., this lack was observed, but at the same time the authors described that a "see-feel" mechanism was quickly developed as an alternative.^34^ A second limitation of robot-assisted microsurgery involves increased costs of the procedure. The MUSA2 robot makes use of reusable conventional microsurgical instruments in the robot, limiting cost and waste of disposables compared to other robotic systems. However, the current literature on robotic assistance with any platform results in increased operative time, both due to the time required to set up the robot and the procedure itself. There is a steep learning curve and with increasing experience more efficient use of the technology is expected.^3^^,^^29^

**Figure 1:**
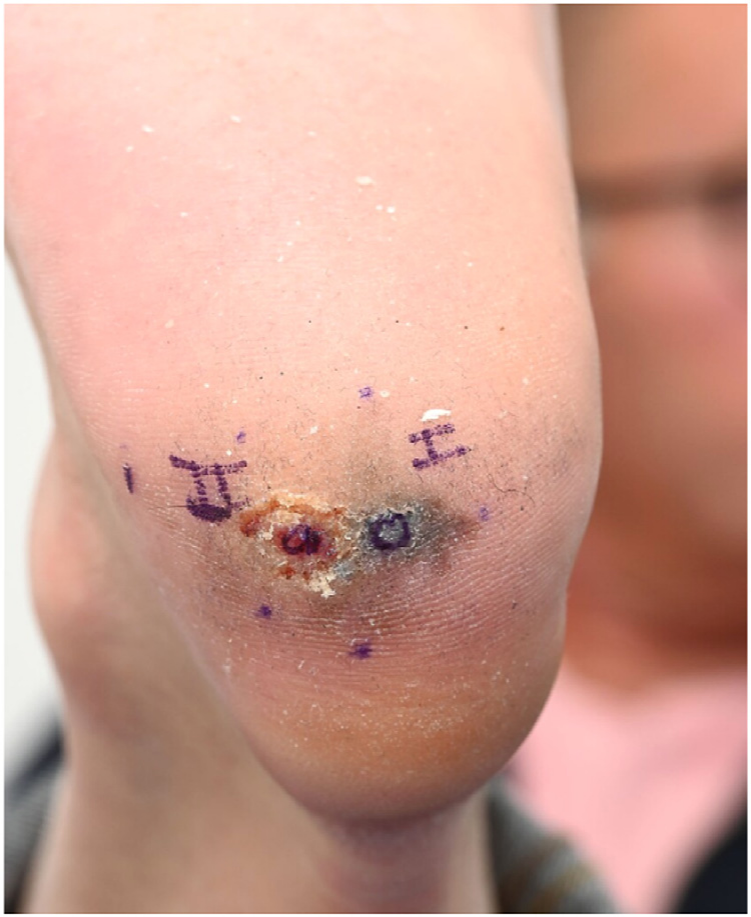
Preoperative view on the melanoma.

**Figure 2:**
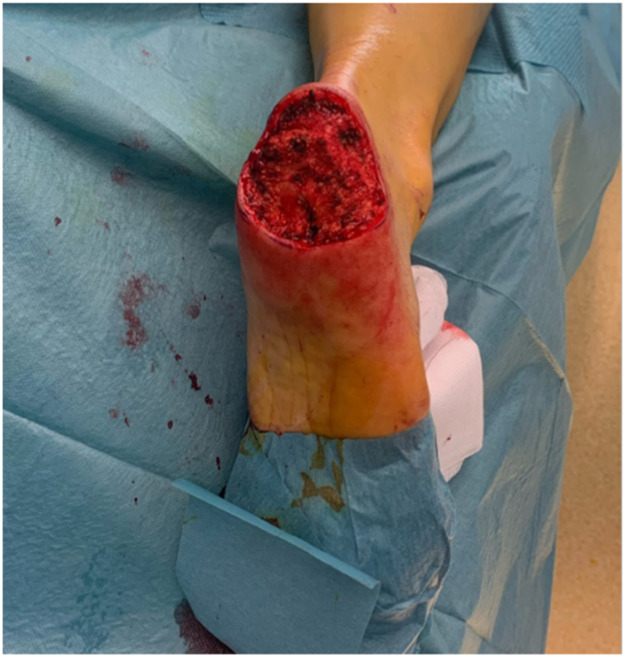
Final defect intraoperative after excision of the melanoma.

**Figure 3:**
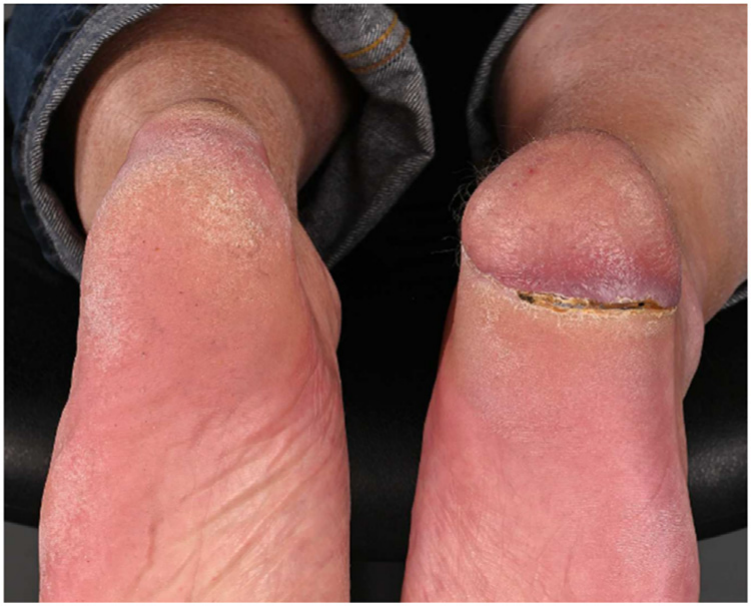
One-year postoperative result.

**Figure 4:**
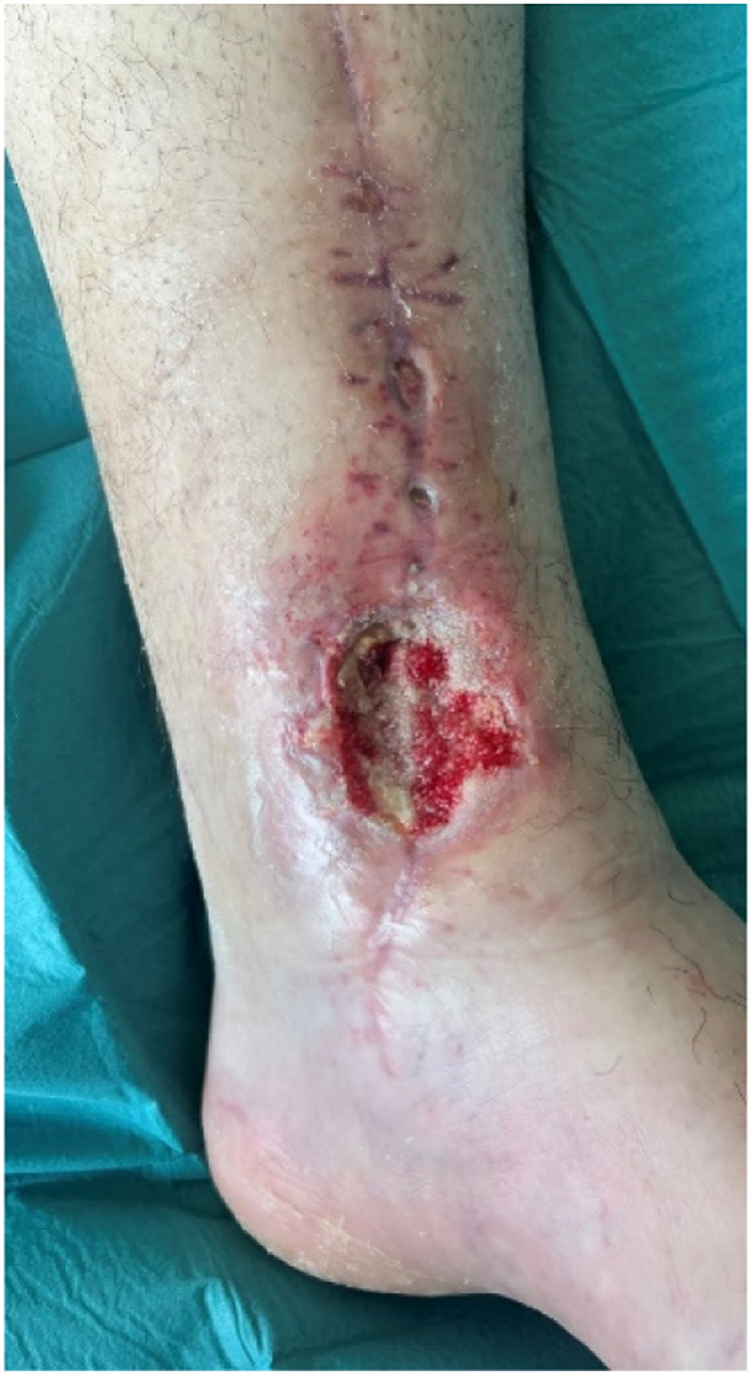
Preoperative defect.

**Figure 5:**
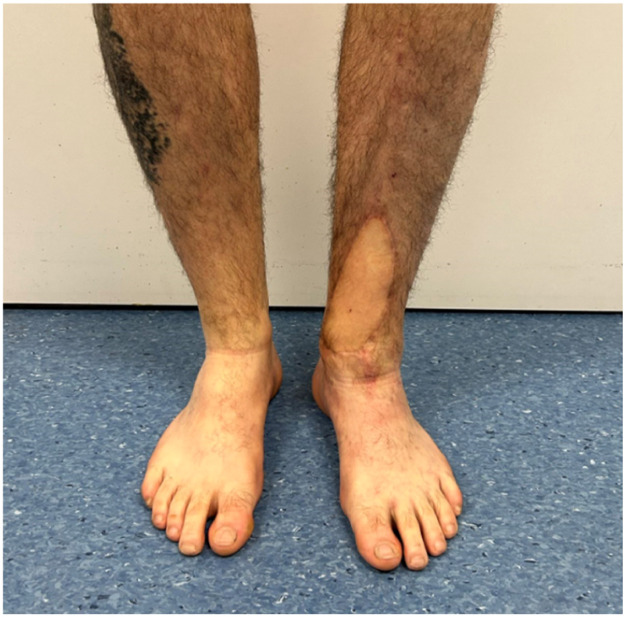
One-year postoperative result.

**Figure 6:**
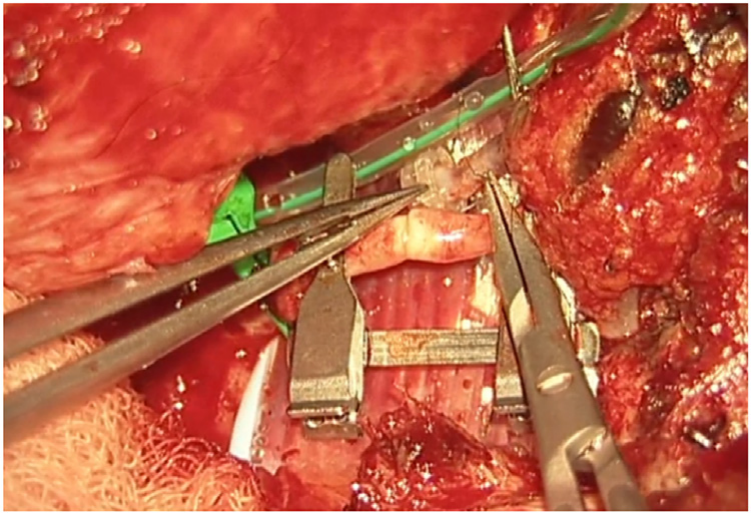
Intraoperative anastomosis positioning.

**Figure 7:**
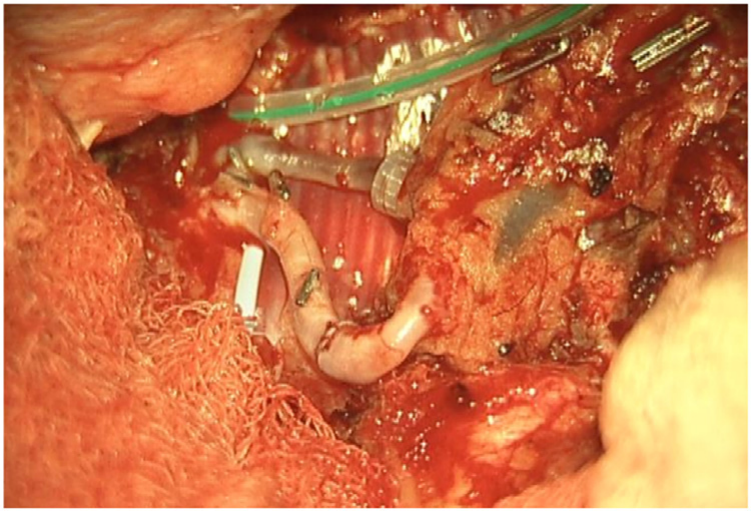
Completed arterial microanastomosis.

**Figure 8:**
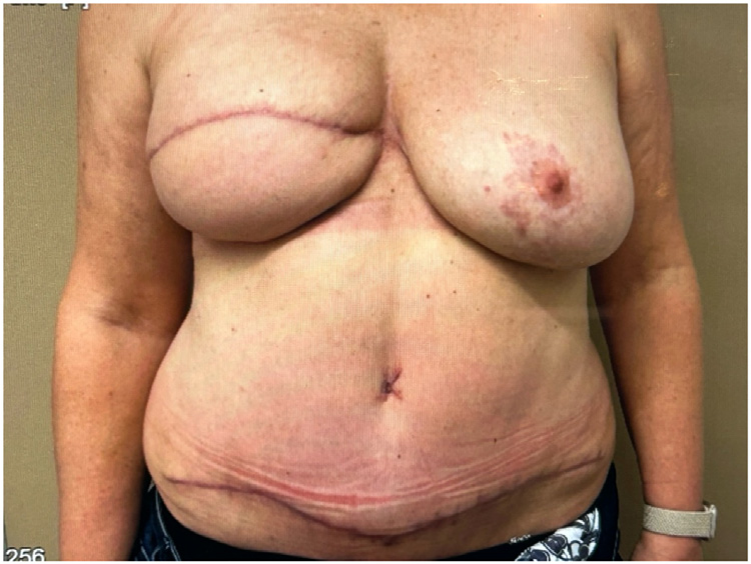
Results at 6 weeks postoperatively.

**Figure 9:**
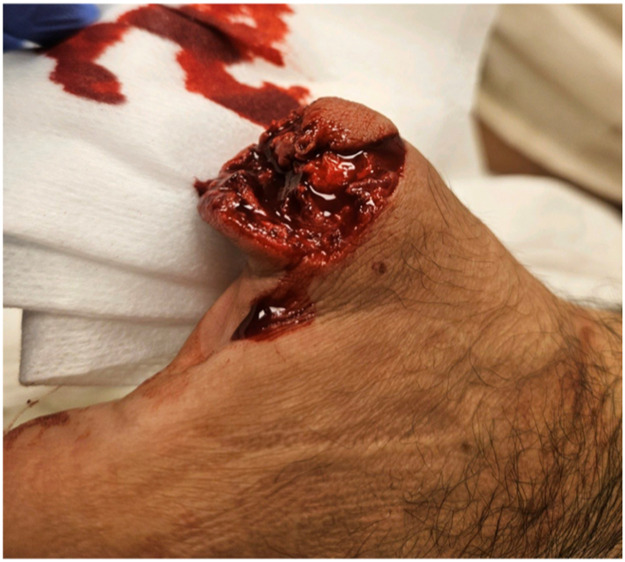
Preoperative defect.

**Figure 10:**
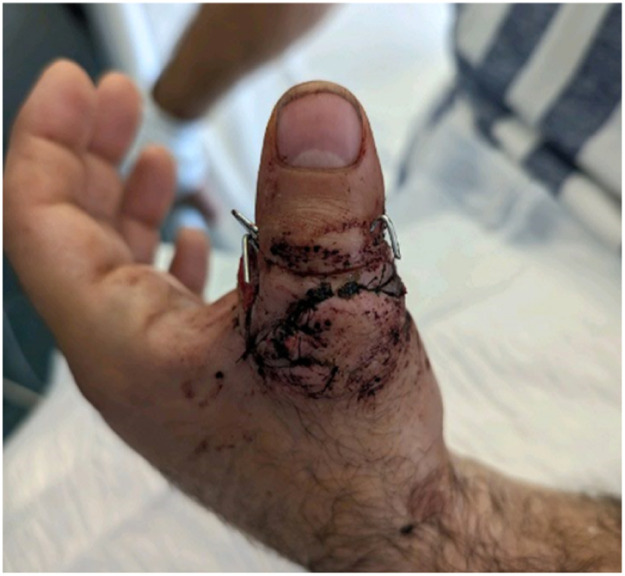
One-day postoperative.

**Figure 11:**
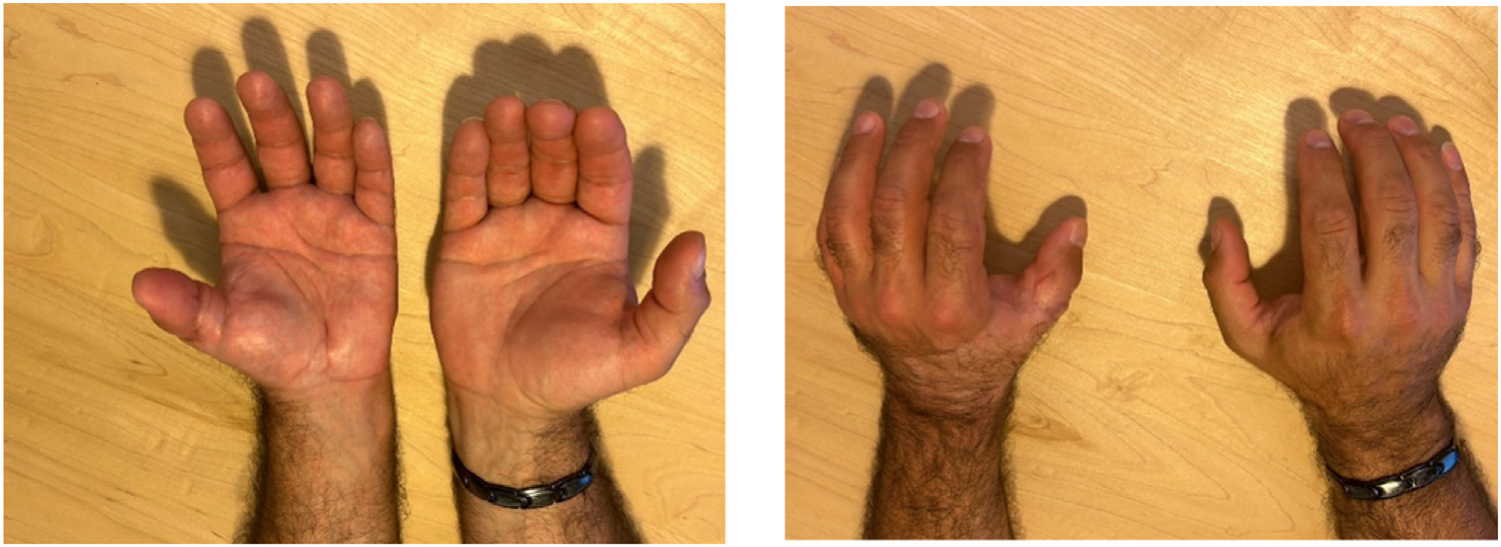
Results at 6-months postoperative.

Despite its limitations, the application of robotic assistance has great potential in microsurgery. Robotic systems can enhance the precision and efficiency of surgical movements and improve ergonomics for the surgical team, an important advantage in lengthy microsurgical procedures, where physical strain is common. The ability to register and provide feedback on forces, motion directions, and speed can be valuable to enhance training in microsurgery. This data can also provide insight in patient care when coupled to patient related outcomes pre- and postoperatively. The advances in artificial intelligence and computing can enable smart robotic systems that can navigate in combination with enhanced imaging systems and eventually enable autonomous tasks in microsurgery. Moving forward, collaboration in multicenter trials with critical and ethical evaluation should guide this path to the future to create safe and better treatments for future patients (Figure 1, Figure 2, Figure 3, Figure 4, Figure 5, Figure 6, Figure 7, Figure 8, Figure 9, Figure 10, Figure 11).

## Ethical approval

These ongoing clinical trials received approval from the Medical Ethics Review Committee (METC) of Maastricht University Medical Center+ and Maastricht University, approval numbers NL60199.068.16/METC162053, NL64178.068.17/METC 172045, NL64506.068.17/METC 172047 and NL81387.068.22/METC22-036, and are conducted in compliance with relevant ethical guidelines and regulations.

## Funding

The robotic system used in these procedures was supplied by MicroSure.

## Declaration of competing interest

Prof. Dr. Van der Hulst is co-founder of and shareholder in MicroSure. Dr. van Mulken is a clinical advisor at and shareholder in MicroSure. The remaining authors have no relevant conflict of interest.
